# Endometrioid adenocarcinoma arising from adenomyosis: two case reports and a literature review

**DOI:** 10.3389/fsurg.2023.1142749

**Published:** 2023-05-05

**Authors:** Jiaying Yang, Qiuying Wang, Xiuchen Han, Qian Liu

**Affiliations:** ^1^ School of Clinical Medicine, Capital Medical University, Beijing, China; ^2^ Senior Department of Obstetrics & Gynecology, The Seventh Medical Center of PLA General Hospital, Beijing, China; ^3^ Department of Obstetric and Gynecology, National Cancer Center/National Clinical Research Center for Cancer/Cancer Hospital & Shenzhen Hospital, Chinese Academy of Medical Sciences and Peking Union Medical College, Shenzhen, China; ^4^ Department of Pathology, The Fourth Medical Center of PLA General Hospital, Beijing, China

**Keywords:** adenomyosis, malignant transformation, endometrioid adenocarcinoma, long-term follow-up, case report

## Abstract

**Background:**

Endometrioid adenocarcinoma is usually diagnosed by endometrial curettage with a positive rate of 94%, while a hysteroscopic examination can increase the positive rate. Differently, endometrioid adenocarcinoma arising from adenomyosis (EAAFA) is always misdiagnosed, even after endometrial curettage or hysteroscopy. EAAFA is rarely reported.

**Case description:**

We reported two cases of EAAFA with long-term follow-ups of 9–10 years. The two cases were misdiagnosed even if endometrial curettage or hysteroscopy was performed. One case complained of postmenopausal vaginal bleeding with elevated CA199, and she was diagnosed by fast-frozen pathology during hysterectomy, followed by bilateral salpingo-oophorectomy and pelvic lymphadenectomy. In another case, a premenopausal woman with acquired progressive dysmenorrhea was diagnosed as EAAFA by the histopathological result after a transvaginal hysterectomy and had to undergo reoperation. The FIGO stage was IB in both cases. Chemotherapy or radiotherapy was performed after the operation. Patients were followed up for 9–10 years, with no metastasis or recurrence being observed.

**Conclusion:**

The diagnosis of EAAFA is always delayed because of tumor-free eutopic endometrium and negative results of the endometrial curettage or hysteroscopy examination. Fast-frozen pathology of the whole uterus helps diagnose EAAFA precisely and avoids reoperation. Adenocarcinoma foci of EAAFA usually involve the myometrium deeply. A better prognosis of EAAFA should be expected due to good differentiation and negative lymphovascular space invasion.

## Introduction

Endometrioid adenocarcinoma is usually diagnosed by endometrial curettage with a positive rate of 94%, while a hysteroscopic examination can increase the positive rate. Differently, endometrioid adenocarcinoma arising from adenomyosis (EAAFA) is always misdiagnosed, even after curettage or hysteroscopy. EAAFA is rarely reported, and its long-term follow-up is extremely rare (1, 2). EAAFA has its particularity in clinical features, examination, and pathology. We reported two cases of EAAFA that were followed up for 9–10 years.

## Case presentation

Case 1 was of a 67-year-old woman presenting with postmenopausal vaginal bleeding for 1 month and was referred to our hospital. She had a history of acquired progressive dysmenorrhea. She denied any history of hypertension or diabetes mellitus. Here body mass index (BMI) was 23 kg/m^2^. An ultrasound scan revealed a solid mass with a clear margin measuring 3.9 cm × 2.2 cm in the uterine cavity, which included a blood flow signal. The serum carbohydrate antigen (CA) 125 and CA19-9 levels were 70.1 U/ml and 455.93 U/ml, respectively. Hysteroscopy was performed on February 29, 2012, and it revealed that the endometrium was thin and tumor-free. A little endometrium was removed. At the same time, an ultrasound examination was done again and revealed that the anterior uterine myometrium was much thicker than the posterior myometrium (2.4 cm vs. 1.2 cm); a solid mass measuring 3.4 cm × 3.3 cm × 2.6 cm was found in the anterior uterine myometrium. Pathology of the endometrium revealed normal glandular cells, partially papillary without atypia. Because CA199 may be elevated in gastrointestinal tumors, gastroscopy and colonoscopy were performed, and normal results were obtained. Diagnosis of sarcoma of the uterus was suspected, while endometrial cancer was excluded.

**Figure 1:**
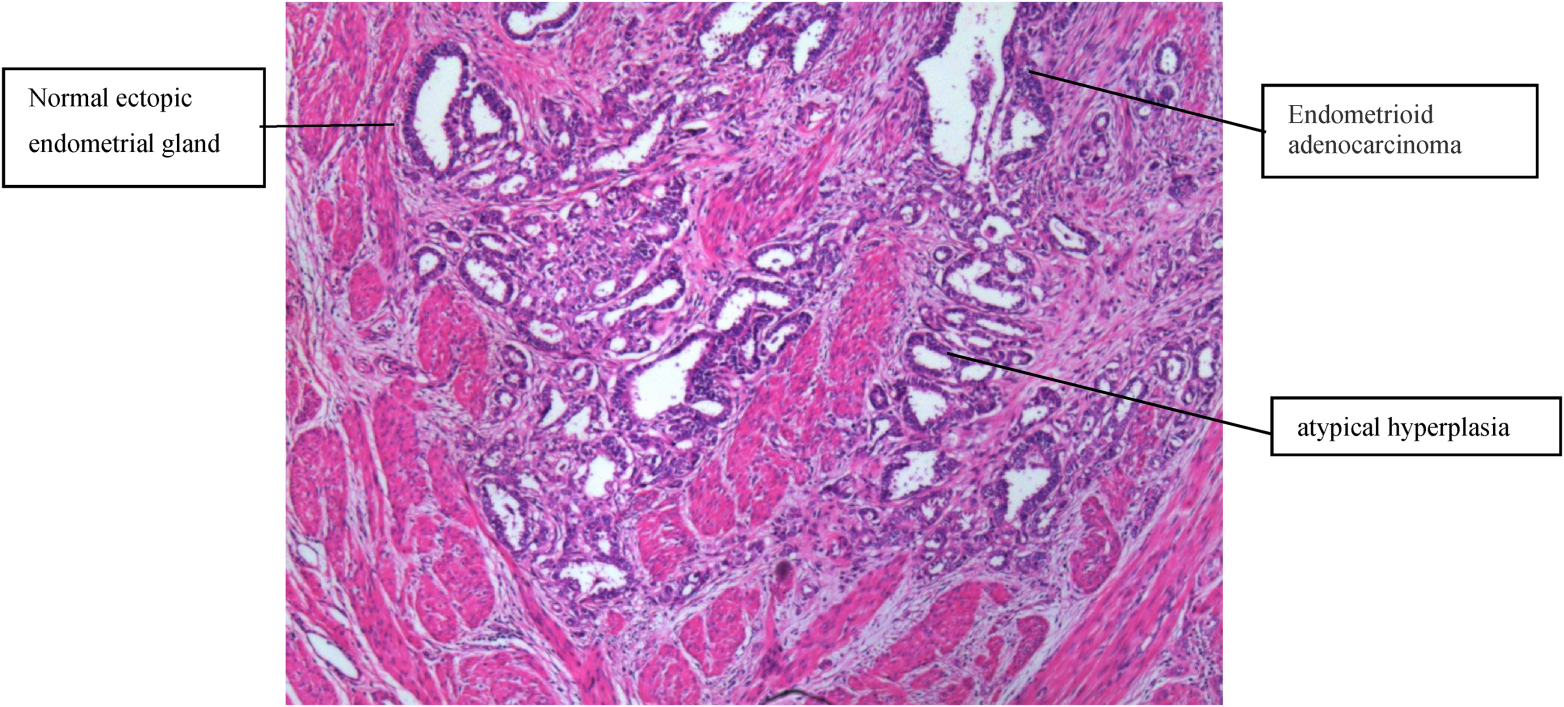
Normal ectopic endometrial glands, atypical hyperplasia, and endometrioid adenocarcinoma were present in the muscular layer (H&E, 100×).

A transabdominal hysterectomy with bilateral salpingo-oophorectomy was performed on March 15, 2012. On gross inspection, the endometrial surface was smooth and tumor-free. On the cut surface, the anterior uterine myometrium was much thicker and harder, with several small scattered lesions. The tumor was clearly apart from the endometrial cavity. Fast-frozen pathology revealed multifocal infiltrating adenocarcinoma tissues within the myometrium. Endometrioid adenocarcinoma was diagnosed. Then, a pelvic lymphadenectomy was performed. The paraffin pathological examination (Figure 1) revealed well-differentiated endometrioid adenocarcinoma, which invaded more than half of the muscle layer, tumor-free eutopic endometrium, ectopic endometrial glands in the myometrium, no metastasis to the adnexa and pelvic lymph node, no cervical invasion, no malignant cells in peritoneal washing fluid, and a positive result of estrogen receptor and progesterone receptor. EAAFA with stage IB was diagnosed definitely. The patient underwent external pelvic radiotherapy and adjuvant chemotherapy with paclitaxel and carboplatin for four cycles. No metastasis or recurrence has been demonstrated with a long-term regular follow-up of 10 years.

**Figure 2:**
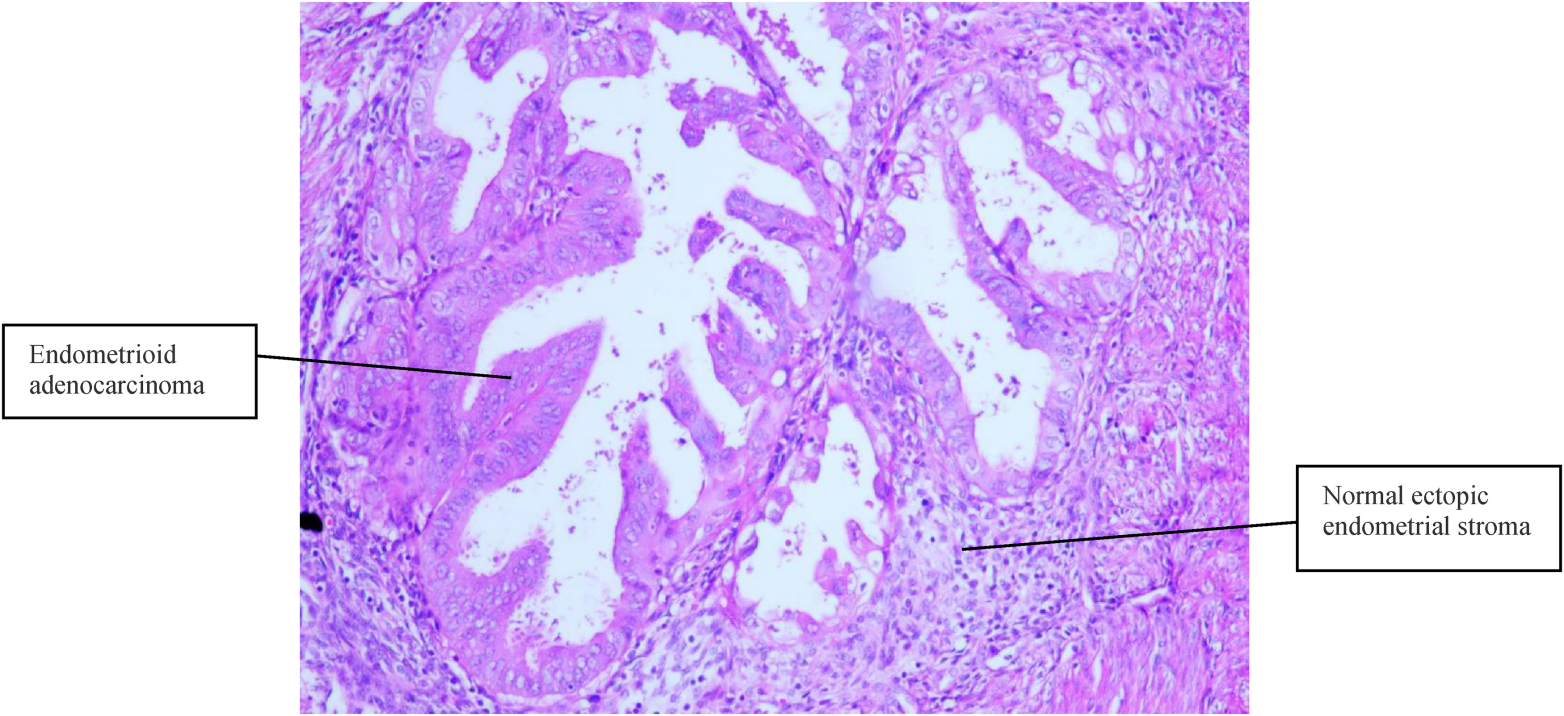
Normal ectopic endometrial stroma and endometrioid adenocarcinoma cells were present in the muscular layer (H&E, 100×).

**Figure 3:**
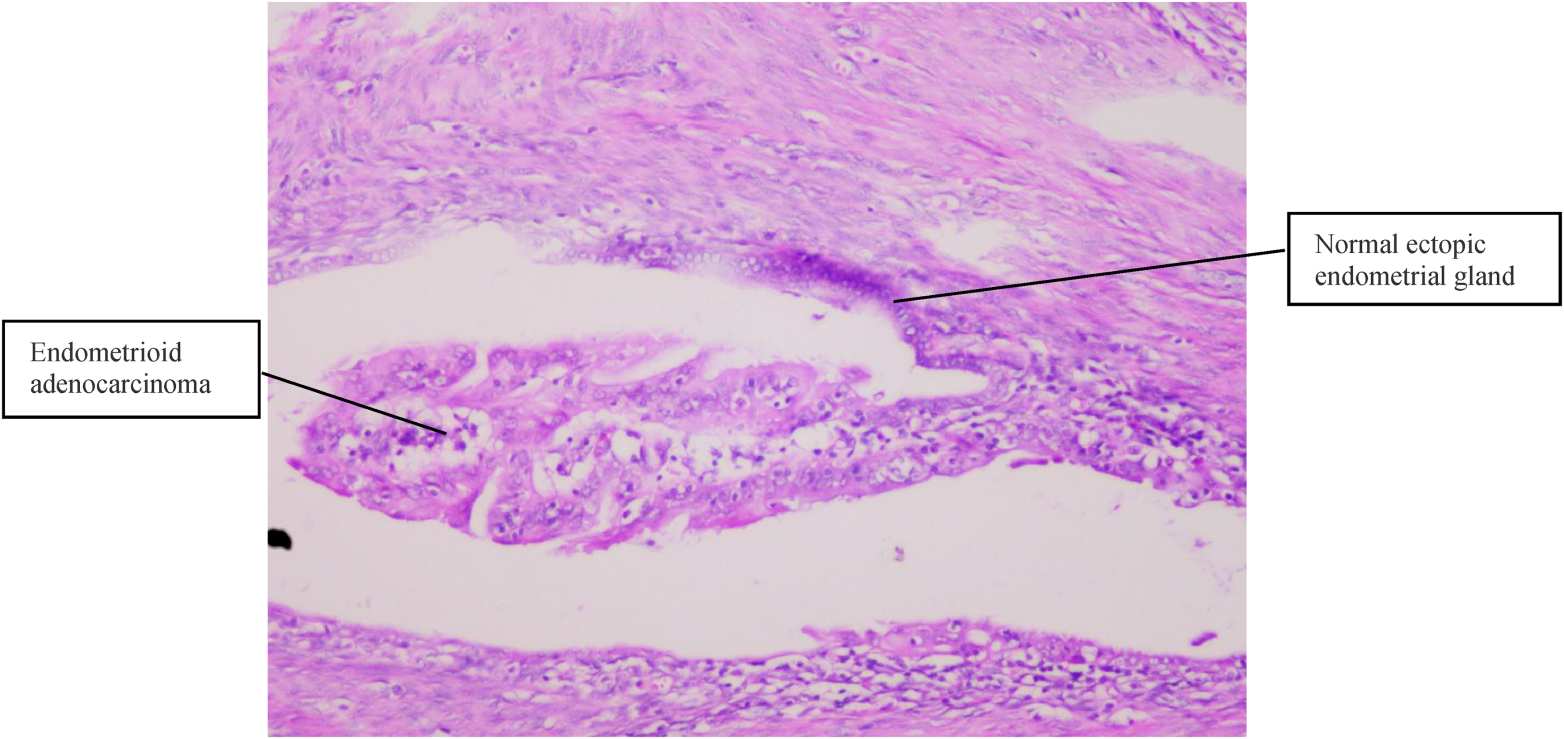
Normal ectopic endometrial glands and endometrioid adenocarcinoma cells were present in the muscular layer (H&E, 100×).

Case 2 was of a 50-year-old premenopausal woman who complained of acquired progressive dysmenorrhea. She was diagnosed with adenomyosis for 7 years. No history of hypertension and diabetes mellitus was reported. Her BMI was 19 kg/m^2^. A transvaginal ultrasound scan revealed an enlarged uterus, uneven echoes in the uterine myometrium, a much thicker anterior wall than the posterior wall of the uterus (5.0 cm vs. 1.7 cm), and a depth of the endometrium of 0.6 cm. The serum CA125 and CA19-9 levels were within normal limits. A gynecologic examination found nothing but an enlarged uterus, the same as 8 weeks of gestation. Endometrial curettage and transvaginal hysterectomy were performed on November 06, 2013. Fast-frozen pathology of endometrial tissue revealed the hyperplasia phase of the endometrium. On gross inspection, the uterus was slightly enlarged like 8 weeks of gestation, the uterine wall was thick, dense, and hard, and multiple coffee-colored bleeding foci were scattered in the myometrium. Histopathological results (Figures 2, 3) showed well-differentiated endometrioid adenocarcinoma invading more than half of the muscle layer, tumor-free eutopic endometrium, endometrial glands and stroma presenting within the myometrium, transitions between benign and malignant glandular structures within the myometrium, and no cervical invasion. The immunohistochemical levels of CD10, SMA, and CA19-9 were negative, while those of ER, PR, and CA125 were positive. The diagnosis of EAAFA with staging IB was supported. Then, secondary surgery of laparoscopic bilateral salpingo-oophorectomy and retroperitoneal lymphadenectomy was performed on November 20, 2013. The paraffin pathological examination revealed no metastasis to the adnexa and the pelvic lymph node. External pelvic radiotherapy was performed following surgery. Her clinical course has been uneventful for 9 years.

## Discussion

Malignant changes in endometriosis are often reported, but those in adenomyosis are rare. Because the incidence of EAAFA of rare, the character of the disease is not well concluded in the published works. We reported two cases of EAAFA with a long-term follow-up of 9–10 years, reviewed the published papers, and tried to conclude the characteristics of the disease.

### Pathological criteria for EAAFA

Sampson's criteria and Scott's complementary criteria were proposed for diagnosing malignant transformation of adenomyosis. Sampson's criteria (3) are as follows: (i) evidence of pre-existing adenomyosis at the site of the malignant lesion; (ii) presence of glandular cells and/or endometrial stromal cells supporting a diagnosis of adenomyosis; (iii) evidence of transitions between the benign and malignant glandular structures; and (iv) absent of carcinoma invasion or metastasis from another source. Scott’s complementary criteria (4) are as follows: presence of transitions between the endometrial epithelium of adenomyosis, borderline malignancy (noninvasive), and invasive carcinoma in the uterine myometrium. Colman (5) modified Sampson's criteria by adding, “the cancer must be absent from eutopic endometrium.” The pathological findings of the two case reports fulfilled the criteria for diagnosing endometrial cancer arising from adenomyosis.

### Morbidity and clinical features

Malignant transformations of endometriosis occur in about 1% of women, but those in adenomyosis are rare, and the frequency has not been reported. Rolly published the first report on EAAFA in 1897. In our review, about 70 cases of malignant tumors arising from adenomyosis have been documented (1, 2, 6–15). Most occurred in postmenopausal women but rarely in premenopausal women (1). We reported two cases of EAAFA, one of which was in a premenopausal woman.

Specific clinical features of EAAFA are lacking. Symptoms are commonly reported as postmenopausal vaginal bleeding, abnormal uterine bleeding, menorrhagia, dysmenorrhea, and anemia. For the two cases in our paper, postmenopausal vaginal bleeding and progressive dysmenorrhea were clinical presentations, respectively. The patient with primary endometrioid carcinoma is often complicated with hypertension, diabetes, or obesity. However, the relationship between EAAFA and hypertension, diabetes, or obesity was not found in our paper, which is consistent with the literature.

### Examination

Endometrial curettage and hysteroscopy are often used to diagnose primary endometrioid adenocarcinoma. Unfortunately, EAAFA is developed by malignant changes in the ectopic endometrium in the uterine myometrium, while the eutopic endometrium is tumor-free, so endometrial curettage and hysteroscopy examination can find nothing. In our paper, endometrial curettage or hysteroscopy was performed, and we found nothing in the uterus cavity. One case was diagnosed by fast-frozen pathology during the operation; another was diagnosed by paraffin pathology after the operation, and the patient had to undergo a second surgery. After reviewing the published papers, we found that cytological tests of the endometrium were negative in almost all cases of EAAFA and misdiagnosis often occurred (7). The eutopic endometrium may be involved when developing to advanced stage (12). Boes et al. (7) reported one patient with EAAFA; the first hysteroscopy examination found nothing, but symptoms still existed; then, the second hysteroscopy examination was performed 1 year later, and pathological examination revealed adenocarcinoma finally.

Tumor markers may help diagnose EAAFA. In the first case of the paper, an increased serum CA-199 (489.89 U/L) level prompted the patient to undergo surgery. Tumor markers, CA125/CA199, were within normal limits or slightly elevated in almost all cases of adenomyosis. If the value of CA125 or CA199 increases in postmenopausal women diagnosed with adenomyosis, malignant changes of adenomyosis should be taken into account. We should be reminded that a regular test of serum CA125 or CA199 is needed for postmenopausal women diagnosed with adenomyosis. CA125 or CA199 is useless in early diagnosis of eutopic endometrium adenocarcinoma, while it may be helpful in following up and evaluating prognosis for the advanced stage.

Magnetic resonance imaging (MRI) may be useful for the diagnosis of EAAFA in the absence of positive pathological results of the endometrium (9). A 41-year-old Japanese woman was observed every 6 months after being diagnosed with adenomyosis. Although she went through menopause at age 51, she occasionally complained of abnormal genital bleeding. The results of endometrial cytology were negative several times. Pelvic MRI demonstrated replacement of the adenomyotic lesion by a poorly demarcated lesion, compared to the findings of prior MRI scanning. Eleven years after the initial diagnosis, endometrial cytology revealed the presence of malignant cells. The report demonstrated the natural course from adenomyosis to adenocarcinoma by MRI. In this patient, periodic MRI evaluations, in conjunction with pathological examinations, played an important role. These facts indicate that MRI with contrast enhancement will be useful for detecting the malignant transformation of adenomyosis.

### Histological subtypes

Adenomyosis commonly undergoes a malignant transformation into endometrioid adenocarcinoma, seldom into clear cell, serous, and poorly differentiated adenocarcinoma (2, 10, 15, 16). Kucera et al. (11) reported that moderately or well-differentiated adenocarcinoma was found in five cases of EAAFA and poorly differentiated in just one case. The differentiation was well in the two cases in our paper, with positive ER and PR.

### Differential diagnosis

EAAFA must be distinguished from endometrium adenocarcinoma coexisting with adenomyosis (EACA). EAAFA is rarely reported. As a result of malignant transformation from uterine adenomyosis, EAAFA usually involves the myometrium deeply with the tumor-free endometrium. In the early stage of EAAFA, the histological examination revealed endometrioid adenocarcinoma located at least 0.5 cm from the endometrium, and the endometrium was without any tumor (8). At the same time, there were transitions between the benign and malignant glandular structures. In the advanced stage of EAAFA, adenocarcinoma cells arising from the ectopic endometrium in the uterus muscle gradually invade the eutopic endometrium (from outside to inside); then, the histological examination has a positive result by hysteroscopy or endometrial curettage. By contrast, EACA is a common finding. For a patient with EACA, an adenocarcinoma cell arises from the eutopic endometrium and invades pre-existing adenomyosis in the muscle layer from the endometrium (from inside to outside) (1). So, the earlier staging and better prognosis are more common in EACA than in EAAFA (17).

### Treatment and prognosis

Because of the rarity of the disease, the standardized therapy of EAAFA is not well characterized yet. EAAFA is commonly treated according to the endometrial adenocarcinoma guidelines. Arising from adenomyosis within the uterine muscle layer, adenocarcinoma foci of EAAFA usually involve the myometrium deeply. Myometrial invasion of EAAFA occurs at a greater depth than endometrial adenocarcinoma, so the FIGO stage is often IB (18). Meanwhile, the negative result of endometrial curettage and hysteroscopy examination delays the diagnosis of EAAFA. In view of the above risk factors, adjuvant chemotherapy and/or pelvic radiotherapy is usually performed after surgery. Both cases in this article were stage IB and underwent radiotherapy or chemotherapy. The reviewed published papers recommend four to six courses of adjuvant combination chemotherapy with paclitaxel and carboplatin (12). However, Taneichi et al. (19) found that deep myometrial invasion of EAAFA did not affect the recurrence or mortality rate. Two cases with stage IB in this paper were followed up for as long as 9–10 years, with no metastasis or recurrence being observed, which was consistent with the study of Taneichi. We think that EAAFA has a more favorable prognosis due to well differentiation and negative lymphovascular space invasion. Different from endometrium adenocarcinoma in stage IB, which invades into deep myometrium from the eutopic endometrium, EAAFA arises from the adenomyotic epithelium within deep myometrium without invasion to surrounding tissues. However, further accumulation of the cases and molecular investigation are needed (17).

## Conclusion

To be emphasized, the diagnosis of EAAFA is always delayed because of a tumor-free eutopic endometrium and a negative result of endometrial curettage or hysteroscopy. It is difficult to diagnose EAAFA preoperatively, so reoperation is often performed, which may be avoided by fast-frozen pathology of the whole uterus during surgery. Despite the deep myometrial invasion of EAAFA, a better prognosis of EAAFA should be expected due to good differentiation and negative lymphovascular space invasion.

## Data Availability

The original contributions presented in the study are included in the article, further inquiries can be directed to the corresponding author.
